# Real-world treatment patterns and clinical outcomes with tucatinib-based therapy in patients with HER2-positive metastatic breast cancer: analyses of two nationwide administrative health claims databases

**DOI:** 10.1007/s10147-025-02800-7

**Published:** 2025-08-06

**Authors:** Carey Anders, Edward Neuberger, Naomi R. M. Schwartz, Karen Bartley, Shu Wang, Yutong Liu, Brian T. Pittner, Peter A. Kaufman, Jane Meisel

**Affiliations:** 1 Duke Cancer Institute (Breast Clinic), Durham, NC USA; 2 Pfizer Inc, South San Francisco, CA USA; 3 Genesis Research, Hoboken, NJ USA; 4 Medical Center (Hematology and Oncology), University of Vermont, Burlington, VT USA; 5 Winship Cancer Institute, Atlanta, GA USA

**Keywords:** HER2+, Metastatic breast cancer, Tucatinib, Trastuzumab deruxtecan, Real-world

## Abstract

**Purpose:**

To describe real-world characteristics and clinical outcomes among patients with HER2+ MBC receiving tucatinib-based treatments.

**Methods:**

This retrospective study included patients diagnosed with HER2+ MBC between January 2017 and December 2022 from two administrative health claims databases, Merative^TM^MarketScan^®^ and the Komodo Healthcare Map^™^. Patient characteristics were captured at baseline (≤ 6 months prior to tucatinib initiation). Outcomes were assessed starting from tucatinib-based treatment initiation and included real-world time to discontinuation (rwTTD) and treatment persistence.

**Results:**

There were 150 patients in MarketScan^®^ who received tucatinib-based therapy: median (IQR) prior lines of therapy (LOT) was 2 (2–4) and 110 patients (73.3%) had brain metastases. 436 patients in Komodo^™^ received tucatinib-based therapy: median (IQR) prior LOTs were 2 (1–3), and 307 (70.4%) had brain metastases. Median (95% CI) rwTTD was 7.4 (5.0–13.1) months in MarketScan^®^ (median follow-up 9.7 months) and 9.0 (7.4–9.8) months in Komodo^™^ (median follow-up 10.3 months). In patients who received tucatinib in combination with trastuzumab and capecitabine immediately following trastuzumab deruxtecan (T-DXd) and after ≥ 2 prior HER2-directed therapies (MarketScan^®^: n = 26, median prior LOT 4; Komodo: *n* = 34, median prior LOT 3), median (95% CI) rwTTD was 5.5 (3.4–not reached [NR]) months in MarketScan^®^ and 4.8 (3.2–NR) months in Komodo.

**Conclusion:**

These results reinforce the real-world effectiveness of tucatinib in patients with HER2+ MBC, including patients with prior T-DXd treatment. Further research is needed to determine the optimal treatment sequencing for patients with HER2+ MBC.

## Introduction

Breast cancer (BC) is the most commonly diagnosed cancer among women and is a leading cause of cancer death, with an estimated 2.3 million new cases worldwide in 2020 [1]. Human epidermal growth factor receptor 2 (HER2) overexpression and/or amplification is present in approximately 15% to 20% of metastatic breast cancers (MBCs) [2–4], and HER2-positive metastatic breast cancer (HER2+ MBC) has historically been associated with an aggressive course of disease, high rates of recurrence, and poor overall survival (OS) [5]. Furthermore, over the course of their disease, up to 50% of patients with HER2+ MBC will develop brain metastases, which is associated with an even poorer prognosis [6, 7].

The treatment landscape for HER2+ MBC is rapidly evolving, with multiple new HER2-targeted therapies emerging in recent years that have led to improvements in clinical outcomes [8]. Despite improvements in outcomes with HER2-targeted therapies, progression-free survival (PFS) and OS still decline at each new line of therapy (LOT), with OS reported to be up to 2 years shorter for third-line (3L) compared with first-line (1L) therapies [9, 10]. Poorer survival outcomes in each subsequent LOT should be taken into consideration when assessing efficacy and effectiveness of new HER2-targeted therapies received in later LOTs.

Tucatinib is a highly selective tyrosine kinase inhibitor (TKI) of the HER2 receptor that has minimal off-target inhibition of the epidermal growth factor receptor (EGFR) [11, 12]. This approval was based on the pivotal randomized, double-blind, placebo-controlled HER2CLIMB trial, in which patients treated with tucatinib in combination with trastuzumab and capecitabine had superior survival outcomes compared with those who received placebo in combination with trastuzumab and capecitabine [13]. Notably, this survival benefit was also observed among patients with active brain metastases, a population that faces a particularly poor prognosis [13, 14]. Consequently, tucatinib is now approved in multiple countries for use in combination with trastuzumab and capecitabine for treating adult patients with HER2+ MBC, including those with brain metastases [15], and is part of treatment guidelines. NCCN guidelines for treatment of HER2+ MBC include tucatinib in combination with trastuzumab and capecitabine, or the antibody drug conjugate trastuzumab deruxtecan (T-DXd), in the second-line (2L, with T-DXd as the preferred therapy in this line) with tucatinib in combination with trastuzumab and capecitabine the category 1, preferred option in 3L, replacing previous standard of care options (including trastuzumab plus capecitabine and lapatinib plus capecitabine) [16]. ASCO and ESMO guidelines recommend tucatinib in combination with trastuzumab and capecitabine in 3L, with ESMO guidelines also indicating it as the preferred treatment in 2L for patients with brain metastases [17, 18].

Because of the rapidly evolving treatment landscape, there is a need to understand real-world treatment patterns and outcomes for patients receiving later-line HER2-targeted therapies. A recent retrospective study evaluating patients with HER2+ MBC in the nationwide deidentified electronic health record–derived Flatiron Health Metastatic Breast Cancer database found that clinical outcomes in the real-world setting were similar to those in HER2CLIMB [19]. Owing to the timing of the trial, HER2CLIMB did not include patients previously treated with T-DXd, and there remains a need for data on the real-world effectiveness of tucatinib-based regimens after treatment with T-DXd.

The aim of this study was to describe characteristics, treatment patterns, and clinical outcomes among patients with HER2+ MBC receiving tucatinib-based treatments in the real-world setting in two nationwide claims databases.

## Materials and methods

### Study design and data sources

This retrospective study was conducted using two nationwide claims databases in the United States: Merative^™^ MarketScan^®^ research database [20] and the Komodo^™^ Healthcare Map^™^ database [21]. MarketScan^®^ and Komodo^™^ are deidentified, aggregated claims datasets providing real-world, patient-level data on 273 million patients and > 325 million patients, respectively. While the MarketScan^®^ database sources its data from commercial and Medicare Advantage (part C) plans [20], Komodo^™^ obtains data from multiple sources across payers, providers, hospital records, lab insights, and others; the databases are representative of US commercial, Medicare, and Medicaid insured populations [21]. Institutional review board approval was not required because the study was noninterventional, and only deidentified patient records were used.

### Population

Patients in the MarketScan^®^ and Komodo^™^ databases between January 2017 and December 2022 who met the following criteria were included: female and aged ≥ 18 years; ≥ 1 claim for BC; ≥ 1 claim for HER2-directed therapy on or after the initial BC diagnosis date; ≥ 1 claim for metastatic cancer up to 1 month prior to initial BC diagnosis and any time after initial BC diagnosis; continuous medical and pharmacy enrollment for ≥ 6 months prior to and ≥ 1 month following the date of a claim indicating a metastatic diagnosis; and received tucatinib-based treatment following its US Food and Drug Administration (FDA) approval in April 2020. The index date was the first claim for tucatinib-based treatment following a claim indicating metastatic diagnosis. Patients were required to have ≥ 1 claim for nonhormone systemic treatment indicated for MBC within 90 days after index date.

Patients were excluded if they had ≥ 2 claims for nonbreast primary cancers in the 6 months prior to the date of a claim indicating a metastatic diagnosis or any claims for metastatic cancer > 1 month prior to initial BC diagnosis.

Demographic and clinical characteristics of patients were assessed during the 6-month baseline period prior to and including the date of initiating tucatinib-based treatment. Patients were categorized as having “de novo” MBC if their initial BC diagnosis occurred within ≤ 90 days prior to MBC diagnosis; otherwise, patients were considered as having “recurrent” MBC.

### LOT definitions

The first rule-based LOT began with the first claim for therapy occurring on or after metastatic diagnosis. All treatments that began within 28 days of the first treatment date were considered part of the same LOT. Regimen discontinuation was defined as a treatment gap of ≥ 60 days from the last medication administration date for a regimen. A treatment switch refers to the initiation of a subsequent LOT and was defined as a claim for a new HER2-directed therapy, TKI, or chemotherapy ≥ 29 days after the start date of the previous LOT. Regimen discontinuation date was defined as the last date of administration prior to a gap in therapy, or 1 day prior to treatment switch in such cases. As hormone therapy may be added without evidence of disease progression in many cases, it was not considered when defining LOT.

### Outcomes

The outcomes assessed starting from initiation of tucatinib-based treatment (index date) consisted of treatment patterns, real-world time to discontinuation (rwTTD), and persistence (defined as the proportion of patients with sufficient follow-up who continued receiving tucatinib-based treatment at 6, 12, 18, and 24 months). Treatment outcomes were assessed in the following cohorts: 1) patients who received any tucatinib-based regimen in any LOT (“overall” cohort); 2) patients who received tucatinib in combination with trastuzumab and capecitabine after ≥ 1 prior HER2-directed therapy in the metastatic setting, as indicated in the FDA label (“FDA label” cohort); 3) patients who received tucatinib in combination with trastuzumab and capecitabine after ≥ 2 prior HER2-directed therapies, as indicated in the European Medicines Agency (EMA) label (“EMA label” cohort); and 4) patients who received tucatinib in combination with trastuzumab and capecitabine immediately following T-DXd and after ≥ 2 prior HER2-directed therapies (“post–T-DXd” cohort).

### Statistical methods

Descriptive statistics for continuous variables included median, 95% CIs, and IQR, while frequencies and percentages were reported for categorical variables. Time-to-event analyses were conducted using the Kaplan–Meier method. Patients without recorded events (owing to loss at follow-up or death) were censored at the date of last health plan enrollment.

## Results

### Populations

Of 383,478 patients with ≥ 1 claim for BC in the MarketScan^®^ database and 1,892,524 patients with ≥ 1 claim for BC in the Komodo^™^ database, 150 patients in MarketScan^®^ and 436 patients in Komodo^™^ received tucatinib-based treatment and met the inclusion criteria (Table 1). In the overall cohorts, brain metastases were present at baseline in 110 patients (73.3%) in MarketScan^®^ and 307 patients (70.4%) in Komodo^™^; median (IQR) prior LOTs were 2 (2–4) in MarketScan^®^ and 2 (1–3) in Komodo^™^ (Table 2). In MarketScan^®^, there were 118 patients (78.7%) in the FDA label cohort and 95 patients (63.3%) in the EMA label cohort. In Komodo^™^, there were 276 patients (63.3%) in the FDA label cohort and 200 patients (45.9%) in the EMA label cohort. The proportion of patients with brain metastases and median prior LOTs among patients in the FDA label and EMA label cohorts were similar to the overall cohort (Online resources 1 and 2).

**Table 1:** Study cohort selection and patient attrition

Criteria		
	Merative^TM ^MarketScan^®^	Komodo Healthcare Map^™^
≥ 1 claim for BC	N = 383,478	N = 1,892,524
	N (% of prior step)	
≥ 1 claim for HER2-directed therapy on or after the initial BC diagnosis date	24,519 (6.4%)	99,692 (5.3%)
≥ 1 claim for metastatic cancer up to 1 month prior to initial BC diagnosis and any time after initial BC diagnosis	10,726 (43.7%)	40,161 (40.3%)
≥ 1 claim for tucatinib after April 2020	310 (2.9%)	1,386 (3.5%)
≥ 6 months of continuous medical and pharmacy enrollment prior to the index date and ≥ 1 month (30 days) on and after the index date, with no allowable gap between monthly enrollment periods	180 (58.1%)	548 (39.5%)
Female and aged ≥ 18 years	178 (98.9%)	546 (99.6%)
No claims for nonhormone systemic treatment indicated for MBC within 90 days after index date; ≤ 2 claims for the same nonbreast primary cancer in the 6-month baseline period; and no claims for metastatic cancer 30 to 180 days prior to the initial BC diagnosis	150 (84.3%)	436 (79.9%)

The index date was the first use of tucatinib-based therapy. The enrollment period was January 2017 to December 2022 for MarketScan^®^ and Komodo^™^

*BC* breast cancer; *HER2* human epidermal growth factor receptor 2; *MBC* metastatic breast cancer

**Table 2:** Baseline characteristics for patients with HER2+ MBC with and without brain metastases receiving tucatinib-based treatment in MarketScan^®^ and Komodo^™^

Characteristic	Merative^™^ MarketScan^®^		Komodo Healthcare Map^™^	
	Overall (N = 150)	Post–T-DXd^a^ (N = 26)	Overall (N = 436)	Post–T-DXd^a^ (N = 34)
Age, median (range), years	53.0 (31.0–89.0)	51.0 (42.0–64.0)	55.0 (20.0–87.0)	58.0 (31.0–83.0)
De novo at diagnosis,^b^ n (%)	64 (42.7)	10 (38.5)	241 (55.3)	12 (35.3)
Sites of metastases, n^c,d^ (%)				
Brain	110 (73.3)	13 (50.0)	307 (70.4)	9 (26.5)
Bone	96 (64.0)	22 (84.6)	279 (64.0)	15 (44.1)
Lung	72 (48.0)	20 (76.9)	172 (39.4)	24 (70.6)
Liver	77 (51.3)	19 (73.1)	211 (48.4)	20 (58.8)
Time from metastatic diagnosis to treatment initiation, median (IQR), months	32.4 (18.6–44.9)	45.8 (30.8–52.7)	19.0 (10.0–32.0)	27.0 (20.0–41.0)
Follow-up, median (IQR), months	9.7 (5.3–20.3)	5.5 (2.5–10.0)	10.3 (5.0–18.2)	5.8 (4.3–10.6)
Prior LOTs, median (IQR)	2 (2–4)	4 (3–5)	2 (1–3)	3 (3–4)
Prior treatments for metastatic disease, n (%)				
Trastuzumab deruxtecan	33 (22.0)	26 (100.0)	54 (12.4)	33 (97.1)
Trastuzumab emtansine	87 (58.0)	19 (73.1)	173 (39.7)	28 (82.4)
Trastuzumab	137 (91.3)	26 (100.0)	356 (81.7)	29 (85.3)
Pertuzumab	123 (82.0)	22 (84.6)	325 (74.5)	28 (82.4)

^a^Patients who received tucatinib in combination with trastuzumab and capecitabine immediately following T-DXd and after ≥ 2 prior HER2-directed therapies

^b^Patients were categorized as having “de novo” MBC if their initial BC diagnosis occurred ≤ 90 days of MBC diagnosis/index date; otherwise, patients were considered as having “recurrent” MBC

^c^Not mutually exclusive

^d^For Komodo Healthcare Map™, sites of metastasis at metastatic diagnostic date

*HER2* human epidermal growth factor receptor 2; *LOT(s)* line(s) of therapy; *MBC* metastatic breast cancer; *T-DXd* trastuzumab deruxtecan

#### Patients receiving tucatinib-based treatment immediately following T-DXd

There were 26 patients (17.3%) in MarketScan^®^ and 34 patients (7.8%) in Komodo^™^ in the post–T-DXd cohort (Table 2). Compared with the overall cohort, a smaller proportion of patients in the post–T-DXd cohort had brain metastases (50.0% in MarketScan^®^ and 26.5% in Komodo^™^) (Table 2; Online resources 1 and 2). The median (IQR) number of prior LOTs in the post–T-DXd cohort was 4 (3–5) in MarketScan^®^ and 3 (3–4) in Komodo^™^ (Table 2). The median (IQR) rwTTD of T-DXd in the post–T-DXd cohorts were 5.7 (3.9–9.8) months in MarketScan^®^ and 6.0 (3.5–8.2) months in Komodo^™^.

### Treatment patterns

**Fig. 1:**
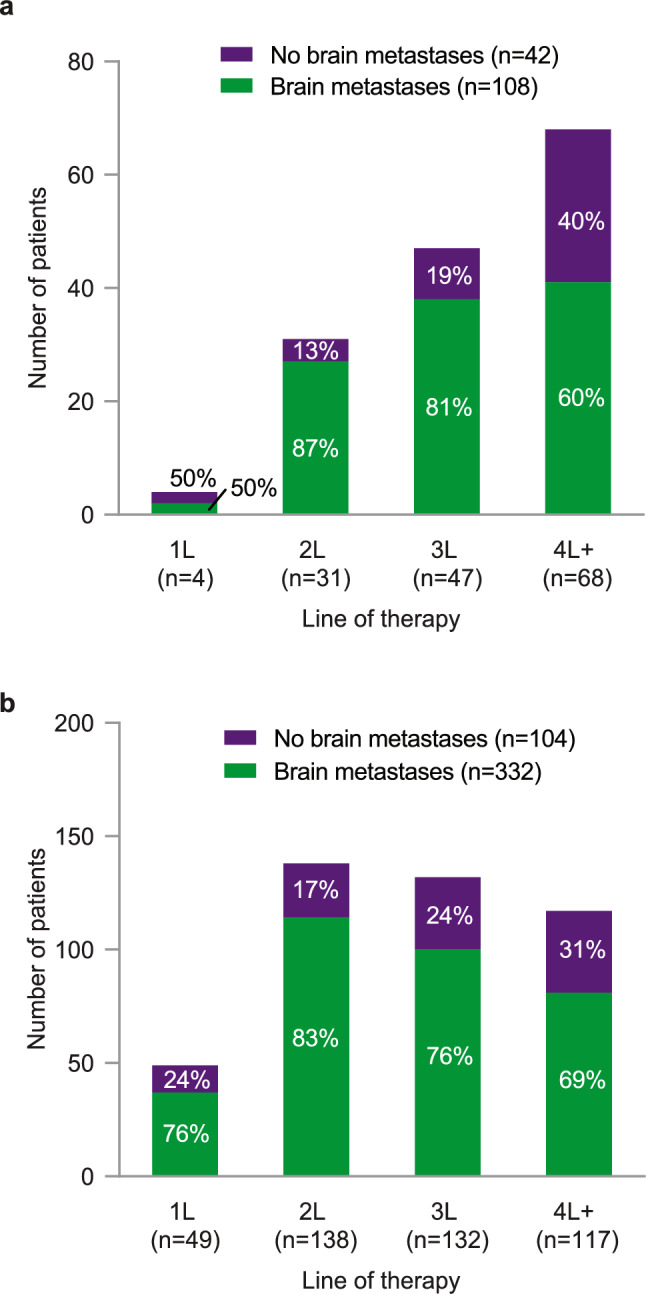
Proportion of patients with and without brain metastases receiving tucatinib-based treatment by LOT in **a** MarketScan^**®**^ and **b** Komodo^™^. 1L, first line; 2L, second line; 3L, third line; 4L +, fourth line or later

In the MarketScan^®^ (N = 150) and Komodo^™^ (N = 436) overall cohorts, tucatinib-based treatment was most commonly received in the 2L or 3L setting (Fig. 1A and B). A larger proportion of patients had brain metastases (compared with those without brain metastases) in all LOTs where tucatinib was received in both MarketScan^®^ and Komodo^™^. In the 2L setting, the most common therapy received prior to tucatinib in both MarketScan^®^ and Komodo^™^ was trastuzumab and pertuzumab–based treatment (*n* = 23 of 31 patients [74.2%] who received tucatinib in 2L in MarketScan^®^; n = 107 of 138 patients [77.5%] who received tucatinib in 2L in Komodo^™^); in 3L, this was T-DM1–based treatment in both MarketScan^®^ and Komodo^™^ (n = 25 of 47 patients [53.2%] who received tucatinib in 3L in MarketScan^®^; n = 63 of 132 patients [47.7%] who received tucatinib in 3L in Komodo^™^). The majority of patients who received tucatinib-based treatment had tucatinib in combination with trastuzumab and capecitabine in both MarketScan^®^ (n = 121, 80.7%) and Komodo^™^ (n = 315, 72.2%). In MarketScan^®^, 29 patients did not receive tucatinib in combination with trastuzumab and capecitabine. Among these, 13 patients (44.8%) received tucatinib with trastuzumab but without capecitabine; 8 patients (27.6%) received tucatinib with capecitabine but without trastuzumab; 7 patients (24.1%) received tucatinib monotherapy; and 1 patient (3.4%) received tucatinib with T-DM1. In Komodo^™^, of patients who did not receive tucatinib in combination with trastuzumab and capecitabine (n = 121), 38 patients (31.4%) received tucatinib with trastuzumab but without capecitabine; 65 patients (53.7%) received tucatinib with capecitabine but without trastuzumab; 15 patients (12.4%) received tucatinib monotherapy; and 3 patients (2.5%) received other combinations of tucatinib.

### rwTTD and persistence

**Fig. 2:**
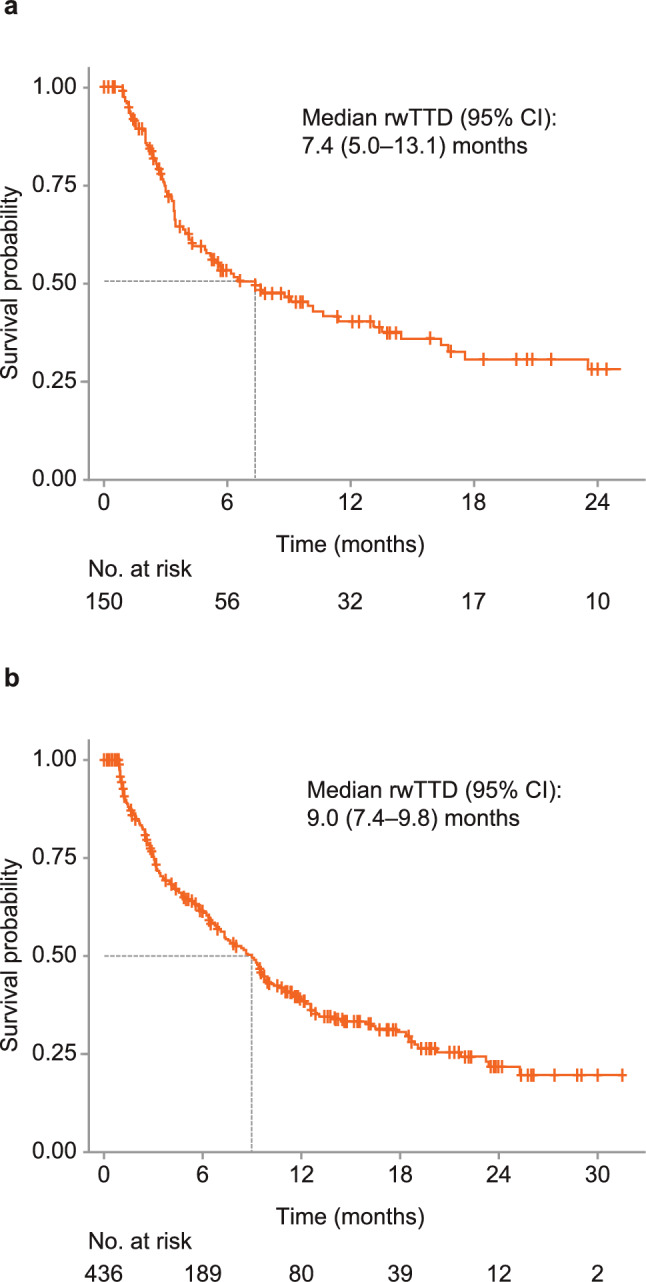
Kaplan–Meier curve for rwTTD among patients receiving tucatinib-based treatment in **a** MarketScan^**®**^ and **b** Komodo^™^. *rwTTD* real-world time to discontinuation

**Fig. 3:**
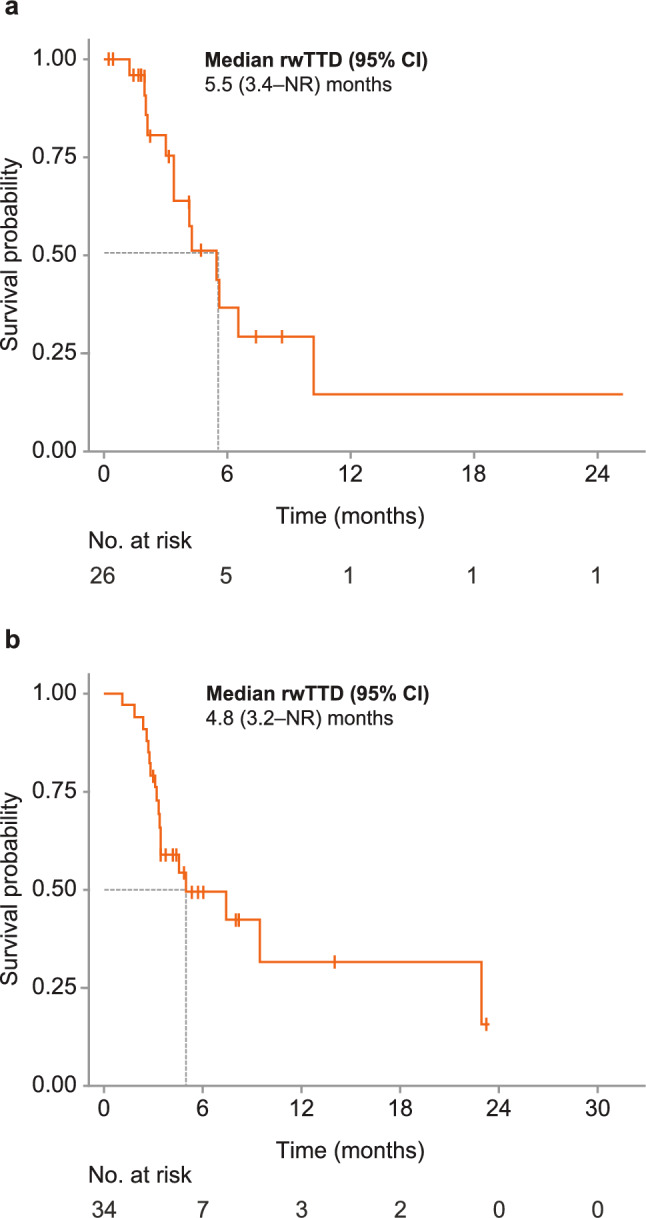
Kaplan–Meier curve for rwTTD among patients who received tucatinib in combination with trastuzumab and capecitabine immediately following T-DXd and after ≥ 2 prior HER2-directed therapies in **a** MarketScan^**®**^ and **b** Komodo^™^. *HER2* human epidermal growth factor receptor 2; *NR* not reached; *rwTTD* real-world time to discontinuation; *T-DXd* trastuzumab deruxtecan

The median (IQR) follow-up from tucatinib-based treatment initiation was 9.7 (5.3–20.3) months in MarketScan® and 10.3 (5.0–18.2) months in Komodo^™^. Median (95% CI) rwTTD in the overall cohort was 7.4 (5.0–13.1) months in MarketScan® and 9.0 (7.4–9.8) months in Komodo^™^ (Fig. 2A and B; Table 3). In the post–T-DXd cohort, median (95% CI) rwTTD was 5.5 (3.4–not reached [NR]) months in MarketScan^®^ and 4.8 (3.2–NR) months in Komodo^™^ (Fig. 3A and B; Table 3). Persistence at 12 months for the overall cohort was 49.2% in MarketScan® and 42.8% in Komodo^™^, while at 24 months persistence was 35.7% in MarketScan^®^ and 26.1% in Komodo^™^ (Table 4). Persistence at 12 months in the post–T-DXd cohort was 20.0% in MarketScan^®^ and 37.5% in Komodo^™^ (Table 4).

**Table 3:** rwTTD for patients receiving tucatinib-based treatment in MarketScan^**®**^ and Komodo^™^

Cohort	Merative^™^ MarketScan^®^	Komodo Healthcare Map^™^
rwTTD, median (95% CI), months		
Overall	7.4 (5.0–13.1)	9.0 (7.4–9.8)
FDA label^a^	10.2 (6.5–16.8)	11.0 (9.3–13.1)
EMA label^b^	9.1 (5.6–16.4)	9.6 (8.1–12.6)
Post–T-DXd^c^	5.5 (3.4–NA)	4.8 (3.2–NA)

^a^Patients who received FDA-approved tucatinib in combination with trastuzumab and capecitabine after ≥ 1 prior HER2-directed therapies in the metastatic setting

^b^Patients who received EMA-approved tucatinib in combination with trastuzumab and capecitabine after ≥ 2 prior HER2-directed therapies

^c^Patients who received tucatinib in combination with trastuzumab and capecitabine immediately following T-DXd and after ≥ 2 prior HER2-directed therapies

*EMA* European Medicines Agency; *FDA* US Food and Drug Administration; *LOT* line of therapy; *NA* not assessed/not reached; *rwTTD* real-world time to discontinuation; *T-DXd* trastuzumab deruxtecan

**Table 4:** Treatment persistence for patients receiving tucatinib-based treatment in MarketScan^**®**^ and Komodo^™^

Cohort	Treatment persistence,^a^ % (n/N)			
	6 months	12 months	18 months	24 months
Merative^™^ MarketScan^®^				
Overall (N = 150)	53.8% (56/104)	49.2% (32/65)	39.5% (17/43)	35.7% (10/28)
FDA label^b^ (n = 118)	61.6% (53/86)	54.5% (30/55)	45.9% (17/37)	43.5% (10/23)
EMA label^c^ (n = 95)	NA	NA	39.4% (13/33)	35.0% (7/20)
Post–T-DXd^d^ (n = 26)	38.4% (5/13)	20.0% (1/5)	NA	NA
Komodo Healthcare Map^™^				
Overall (N = 436)	62.1% (185/298)	42.8% (80/187)	35.1% (39/111)	26.1% (12/46)
FDA label^b^ (n = 276)	71.1% (135/190)	50.8% (64/126)	37.7% (29/77)	22.6% (7/31)
EMA label^c^ (n = 200)	68.1% (94/138)	46.6% (41/88)	32.7% (18/55)	20.0% (5/25)
Post–T-DXd^d^ (n = 34)	43.8% (7/16)	37.5% (3/8)	NA	NA

^a^Persistence is the proportion of patients with follow-up (N) and still on therapy (n) at each timepoint

^b^Patients who received FDA-approved tucatinib in combination with trastuzumab and capecitabine after ≥ 1 prior HER2-directed therapies in the metastatic setting

^c^Patients who received EMA-approved tucatinib in combination with trastuzumab and capecitabine after ≥ 2 prior HER2-directed therapies

^d^Patients who received tucatinib in combination with trastuzumab and capecitabine immediately following T-DXd and after ≥ 2 prior HER2-directed therapies

*EMA* European Medicines Agency; *FDA* US Food and Drug Administration; *LOT* line of therapy; *NA* not assessed/not reached; *T-DXd* trastuzumab deruxtecan

## Discussion

The treatment landscape for HER2+ MBC is dynamic, with several newly approved HER2-targeted treatments since 2020. Tucatinib in combination with trastuzumab and capecitabine was approved for the treatment of HER2+ MBC based on the pivotal HER2CLIMB trial, in which significantly improved PFS and OS were observed in a population of heavily pretreated patients with HER2+ MBC, including in those with brain metastasis [13]. This study provides valuable information on use of tucatinib in real-world patients with HER2+ MBC and adds to the growing body of evidence for this patient group. These findings also offer new insight into the effectiveness of tucatinib in patients previously treated with T-DXd, the preferred 2L therapy for patients with HER2+ MBC [16–18].

The patients included in this analysis had similar characteristics to those enrolled in HER2CLIMB, with the exception of the proportion of patients with brain metastases and treatment history [13]. As was the case in the previously published analysis of the Flatiron electronic health record database, a higher proportion of patients in this study had brain metastases at baseline (73.3% of patients in MarketScan^®^, 70.4% of patients in Komodo^™^, and 70.8% of patients in the Flatiron study) compared with the proportion in HER2CLIMB (48.3%) [13, 19]. Additionally, all patients in HER2CLIMB had been previously treated with pertuzumab and T-DM1, whereas in this study, 58.0% in MarketScan^®^ and 39.7% in Komodo^™^ had prior T-DM1; 82.0% in MarketScan^®^ and 74.5% in Komodo^™^ had prior pertuzumab; and 91.3% in MarketScan^®^ and 81.7% in Komodo^™^ had prior trastuzumab in the metastatic setting.

Despite these differences, clinical outcomes following tucatinib treatment were comparable across the real-world studies and HER2CLIMB: median (95% CI) rwTTD in the overall cohort was 7.4 (5.0–13.1) months in MarketScan^®^; 9.0 (7.4–9.8) months in Komodo^™^; and 6.5 (5.4–8.8) months in the Flatiron study [19], compared with a median duration of exposure of 7.3 months in HER2CLIMB [13].

In this study, tucatinib treatment demonstrated effectiveness among the various patient subgroups evaluated, including those who received tucatinib in combination with trastuzumab and capecitabine as indicated in the FDA or EMA labels, and those who received tucatinib immediately following T-DXd. As data on disease progression were not available, rwTTD with tucatinib-based treatment was used as a marker of tolerability and a conservative estimate of progression [22]. Notably, rwTTD was shorter and persistence was lower in patients who received tucatinib-based treatment immediately following T-DXd; however, these patients were heavily pretreated (median of 3 or 4 prior LOTs for this group, compared with 2 for the overall population).

Median rwTTD for T-DXd prior to tucatinib in this study was only 5.7 months in MarketScan^®^ and 6.0 months in Komodo^™^, compared with 10.0 months in DESTINY-Breast01 [23]. Outcomes from MarketScan^®^ and Komodo are similar to those from a French cohort study of treatment outcomes with tucatinib-based treatment in patients who had previously received T-DXd (median 5 prior LOT) and reported a median (95% CI) PFS of 4.7 (3.9–5.6) months [24]. This suggests that patients in these real-world databases had more aggressive disease and less than expected benefit from T-DXd than those in clinical trials. While there are limited data from clinical trials, these real-world indicate tucatinib is a viable option for patients with prior exposure to T-DXd [19, 24].

The results of this study align with clinical trial evidence and prior real-world studies, reinforcing the durable effectiveness of tucatinib in the real-world setting in patients with HER2+ MBC with and without brain metastases. The consistency between clinical efficacy and real-world effectiveness with tucatinib is noteworthy, as clinical trial outcomes often do not reflect outcomes observed in the real-world setting [25]. This is acknowledged in a concept now described as an “efficacy-effectiveness gap” [25–29], which has been observed with a number of new cancer treatments, including other HER2-directed therapies [30–32]. Further studies will not only contribute to the knowledge base regarding real-world treatment of HER2+ MBC but will also help determine optimal treatment sequencing for patients with HER2+ MBC.

### Limitations

As with all administrative health claims data, these analyses were limited by the potential for missing or incomplete records. In addition, these analyses are unadjusted and purely descriptive in nature, and further stratification of cohorts by both LOT and brain metastases would have resulted in small sample sizes that precluded robust analyses. In this study, it was not possible to confirm to what extent there was overlap between the MarketScan^®^ and Komodo^™^ cohorts, although the data are sourced differently, with MarketScan^®^ sourced from large employer health plans and Komodo^™^ from multiple sources. The findings of any health claims database analysis, including the studies reported here, may not be generalizable to patient populations that are not represented in the databases being evaluated.

As patients may have received tucatinib in later LOTs than the guideline-recommended 2L or 3L setting (owing to a diagnosis prior to tucatinib approval), the outcomes in this study may be more conservative than in current clinical practice. The proportion of patients with brain metastases in this study (approximately 70%) was higher than that reported in a prior real-world study of patients with HER2+ MBC who received any treatment (approximately 30%), which may indicate that patients with brain metastases are more likely to be prescribed tucatinib, given its efficacy in this population [33]. Patients were considered to discontinue on the date of last administration of therapy or prescription fill for oral medications; data on individual reasons for discontinuation were not available. Due to the relatively recent adoption of T-DXd in clinical practice, patients included in the post–T-DXd cohort may be those with shorter durations of T-DXd therapy; therefore, outcomes with tucatinib in this group may not be representative of all patients receiving tucatinib post–T-DXd.

## Conclusions

This real-world analysis utilizing data from two nationwide claims databases, MarketScan^®^ and Komodo^™^, reinforces previously published real-world evidence [19] and results from the pivotal HER2CLIMB trial [13]. These data illustrate the effectiveness of tucatinib in the real-world setting across LOTs for patients with HER2+ MBC with and without brain metastases and suggest that tucatinib therapy is an effective treatment option in accordance with the FDA and EMA labels, including for patients previously treated with T-DXd.

## Data Availability

Data supporting the results of this study are provided within the manuscript or in the online resources. The Merative^™^ MarketScan^®^ and Komodo Healthcare Map^™^ databases are proprietary databases accessible via a subscription.
